# Detection of Sepsis in Platelets Using MicroRNAs and Membrane Antigens

**DOI:** 10.3390/genes12121877

**Published:** 2021-11-25

**Authors:** Priscilla Cristina Moura Vieira Corrêa, Débora Monteiro Carneiro, Luciana do Socorro da Silva Valente, Fabíola Marques Diogo, Leticia Martins Lamarão, Jersey Heitor da Silva Maués, Caroline Aquino Moreira-Nunes, Rommel Mario Rodríguez Burbano

**Affiliations:** 1 Molecular Biology Laboratory, Ophir Loyola Hospital, Belém 66063-240, PA, Brazil; priscillavieira@hotmail.com (P.C.M.V.C.); deboramonteirocarneiro@gmail.com (D.M.C.); letlamarao@hotmail.com (L.M.L.); 2 Foundation Center for Hemotherapy and Hematology of Pará, Belém 66033-000, PA, Brazil; luvalente86@gmail.com; 3 Oncology Research Center, Federal University of Pará, Belém 66073-005, PA, Brazil; fabioladiogo@hotmail.com; 4 Hematology and Transfusion Medicine Center, University of Campinas, Campinas 13083-970, SP, Brazil; jerseymaues@gmail.com; 5 Department of Medicine, Pharmacogenetics Laboratory, Drug Research and Development Center, Federal University of Ceará, Fortaleza 60430-275, CE, Brazil; carolfam@gmail.com

**Keywords:** sepsis, platelets, microRNA, membrane antigens

## Abstract

The present study proposes to legitimize in sepsis a characteristic found in platelets that suffer storage lesions in blood banks, which is the increased expression of miRNA miR-320a in relation to miR-127. Under physiologically normal conditions, an inverse relationship is observed. The aim of this study was to verify whether the analysis of miR-320a and miR-127 expression in platelets could detect a decrease in their viability and function due to the presence of pathogens in the blood of patients hospitalized in the Intensive Care Unit. We also investigated the expression of membrane antigens sensitive to platelet activation. Of the 200 patients analyzed, only those who developed sepsis (140) were found to have a higher relative quantity of miR-320a than that of miR-127. This characteristic and the increased expression of membrane antigens P2Y12, CD62P, CD41, and CD61 showed a significant association (*p* < 0.01) with all types of sepsis evaluated in this study. Additionally, 40% of patients hospitalized for sepsis had negative results for the first cultures. We conclude that analysis of miR-127 and miR-320a expression combined with membrane antigens evaluation, in association with the available clinical and diagnostic parameters, are important tools to detect the onset of sepsis.

## 1. Introduction

The incidence of sepsis varies depending on the hospital studied, being higher in those dealing with more clinically severe patients, such as cancer hospitals. Early diagnosis and treatment are the main weapons for reducing mortality [1]. The pro-inflammatory response triggered during sepsis leads to activation of the coagulation system and endothelial damage, with the activation of platelets that are stimulated by direct interactions with pathogens [2].

Platelets are small anucleated cells derived from the cytoplasmic fragmentation of bone marrow megakaryocytes. The outer membrane of activated (stimulated) platelets integrates glycoprotein antigens (Gps) and differentiation complexes (CDs), many of which function as receptors and interact with procoagulant factors, adhesion, aggregation, and inhibition molecules. Thus, the increased or decreased expression of these membrane biomarkers is a good indicator of platelet activation [3,4,5].

Thrombocytopenia is commonly associated with sepsis and infections, which in turn are characterized by a profound immune response to the invading pathogen. Platelets exert considerable immunological, antibacterial, and antiviral actions, and therefore are active participants in the host’s response. Platelets contribute to the immune response through several mechanisms, including providing the endothelium with a pro-inflammatory phenotype, increasing and amplifying leukocyte recruitment and inflammation, promoting the effector functions of immune cells, and ensuring an optimal adaptive immune response [6].

Despite the lack of nucleus and genomic DNA, platelets have several types of RNA, ranging from messenger RNAs to non-coding RNAs such as microRNAs (miRNAs), all originating from megakaryocytes. In the activation process, platelets are able to use their own translation mechanism to synthesize proteins, suggesting the possibility of a post-transcriptional gene regulation in these cells [7].

The present study proposes to legitimize in sepsis a characteristic found in platelets that suffer storage lesions in blood banks—that is, the increase in the platelet concentration of miRNA miR-320a in relation to the concentration of miR-127. When platelets are not activated (that is, when they are in a physiologically normal stage), the quantity of miR-127 is greater than that of miR-320a. When the platelet concentrate (PC) stored in a blood bank undergoes aging due to excessive storage time or contamination by microbial agents, the degradation of miR-320a is lower than that of miR-127, and as a result miR-320a is present at a higher concentration [8,9,10].

Thus, the aim of this study was to verify whether the analysis of miR-320a and miR-127 expression in platelets could detect a decrease in their viability and function due to the presence of pathogens in the blood of patients hospitalized in Intensive Care Units (ICUs), as well as the association with the expression of membrane antigens sensitive to platelet activation.

## 2. Materials and Methods

A total of 200 patients were evaluated before and after admission to the ICU of the Ophir Loyola Oncological Hospital, a cancer reference in Northern Brazil, from January 2019 to December 2020. Patients with sepsis were classified according to the Sequential Organ Failure Assessment (SOFA) score [11]. The Acute Physiology and Chronic Health Disease Classification System II (APACHE score) estimated mortality scoring system was calculated based on laboratory and clinical data obtained in the first 24 h of ICU admission. This study analyzed the presence of pathogens between 24 and 48 h after the diagnosis of sepsis, to provide a reassessment of the patient, identify the worsening of the pathology, and allow interventions and changes in the therapeutic approach.

The present study was approved by the Ethics Committee for Research on Human Beings of the Hospital Ophir Loyola (approval number 4.071.931). All patients or their guardians were informed in advance about the procedures and the purpose of the research, in addition to reading and signing the informed consent form.

One hundred healthy volunteers were used as negative controls for immunophenotyping and miRNA studies. An anticoagulant tube was centrifuged to remove red cells and obtain platelet-rich plasma. Platelet quantification was performed using a Sysmex XE 5000TM hematology counter. In the complete blood counts, the mean platelet volume (MPV) was also evaluated.

### 2.1. Evaluation of Pathogenic Agents

The culture techniques performed were blood cultures, analysis of ascites fluid, uroculture, abdominal and pleural secretions, cerebrospinal fluid, and sputum and tracheal aspirate. These were performed before starting antimicrobial therapy. Procalcitonin prohormone was also measured using the AQT90 FLEX analyzer (Radiometer, Copenhagen, Denmark).

### 2.2. Platelet Immunophenotyping (Platelet Activation)

Platelet-rich plasma samples were pre-incubated for 15 min at room temperature with 5 µL of one of the following monoclonal antibodies: P2Y12 Platelet ADP Receptor antibody (Sigma-Aldrich, Saint Louis, MO, USA), CD62P/P-Selectin Antibody APC conjugate (Thermo Fisher Scientific^®^, Waltham, MA, USA), Integrin alpha-2b/CD41 Antibody FITC conjugate (Thermo Fisher Scientific^®^, Waltham, MA, USA), and Integrin beta-3/CD61 Antibody APC conjugate (Thermo Fisher Scientific, Waltham, MA, USA), which have specificity for the platelet antigens P2Y12, P-Selectin (CD62P), GpIIb (CD41), and GpIIIa (CD61), respectively. We characterized the basal state of platelet activation, measured by the mean fluorescence intensity (MFI) of the plasma, which means the higher the fluorescence, the greater the labeling of antibodies against the plasma membrane [12]. For each sample submitted to flow cytometry, 10,000 events were analyzed using Attune NxT Software (Thermo Fisher Scientific, Waltham, MA, USA).

### 2.3. Real-Time Polymerase Chain Reaction

Quantification of miRNAs expression in rich plasma and platelets was performed by real-time polymerase chain reaction (RT-qPCR). The miRNA miR-191 was selected as an internal control for reverse transcription efficiency and as a reference miRNA, as it is the most highly expressed in platelets [8]. The miRNAs were reverse transcribed using the TaqMan MicroRNA Reverse Transcription kit, according to the manufacturer’s protocol (Thermo Fisher Scientific^®^, Waltham, MA, USA). The expression quantification of miRNAs miR-127, miR-320a, and miR-191 was measured with TaqMan assays PN002229, PN002277, and PN002678, respectively. Complementary DNA was amplified by RT-qPCR using the TaqMan Universal Master Mix II kit with UNG (Thermo Fisher Scientific^®^, Waltham, MA, USA) in the thermocycler QuantStudio 7 Real-Time PCR Systems (Applied Biosystems^®^, Foster City, CA, USA). All RT-qPCR reactions were performed in triplicate.

### 2.4. Statistical Analysis

Parameters evaluated in patient platelets were tested using the Student’s t-test with Welch correction. Differences were considered significant for *p*-value <0.01. The Shapiro–Wilk test was the non-parametric normality test used to assess the distribution of miRNA expression (miR-127/miR-320a), with *p* < 0.01 considered significant. The normality test was applied only for microRNA expression (miR-127/miR-320a) under all conditions. The Wilcoxon paired-sample test was applied to estimate the significance of expression of miR-127 and miR-320a in relation to miR-191, which was used as an internal control for validation analysis (*p* < 0.01). We used this non-parametric test because the data distribution of expression of these two microRNAs did not follow the normality criteria when tested with Shapiro–Wilk. Unsupervised clustering and heatmaps were built with the heatmap.2 function. Heatmaps were used to show hierarchical groupings of the gene expression profile of the samples, using the Z-score metric. All statistical analysis was performed using R (https://www.r-project.org, accessed on 4 October 2021).

## 3. Results

Of the 200 patients admitted to the ICU, 140 had sepsis of different severity, with a mortality of 78.6% and mean APACHE II score of 35. Platelet counts were higher in surviving patients than in non-survivors. An increased mean platelet volume (above 11 phentoliters) was found in patients who progressed to sepsis.

Table 1 shows the general characteristics of the patients studied from 2019 to 2020. Of the patients admitted to the ICU, 122 (61%) were male. The mean age of patients was 60.75 years (ranging from 28 to 89 years). The average length of stay in the ICU for patients with sepsis was 15.7 days, while the average for those without sepsis was 5 days.

Most admissions to the ICU (66%) were for medical reasons and only 34% for surgery. The main focus of infections was pulmonary (65%), followed by abdominal (30%) and urinary bacteremia (5%). The most frequent comorbidities were neoplasms (80%) (as this study was carried out in an oncology hospital), diabetes mellitus (10.7%), and systemic arterial hypertension (9.3%). As for invasive procedures, most patients underwent central vascular catheterization (82.9%), mechanical ventilation (79.3%), and tracheostomy (75%).

### 3.1. Types of Sepsis

Of the 140 patients with sepsis, 18 (12.9%) had uncomplicated sepsis, 22 (15.7%) had severe sepsis, and 100 (71.4%, *p* < 0.05) developed septic shock. As for the main risk factors, 79 patients with sepsis were older than 65 years (56.4%) and all progressed to death, showing that a higher age favored the susceptibility of these patients.

In total, 110 (78.6%) died regardless of the worsening of the disease. As expected, the greater the aggravation of this pathology, the longer the hospital stay. In addition, the greater the severity of sepsis, the greater the exposure to invasive procedures, thus all patients with septic shock underwent some invasive procedure during their stay in the ICU, mainly through a central vascular catheter, urinary catheter, mechanical ventilation or tracheostomy, which may explain the worsening of sepsis (Table 1).

### 3.2. Sepsis Etiology

According to the worsening of the disease, bacteria were the main etiological agents (75%), of which Gram-negative bacilli were the most frequent (26%), especially in patients with greater disease severity. From this group, the non-fermenting bacilli (58%) and the *Enterobacteriaceae* family (52%) stand out. Gram-positive cocci represented 7% of the total of isolated microorganisms, of which 60% were *Staphylococcus aureus*, 20% *Enterococcus* sp., and 20% coagulase-negative *Staphylococcus*. Furthermore, in 67% of cases, other bacteria were also found, such as mycobacteria (69%), *Clostridium tetani* (19%), and *Neisseria meningitidis* (12%). Viruses and fungi accounted for 90% of the samples, with COVID-19 being the most frequent (92%), while HIV, H1N1 virus, and *Candida albicans* yeast were found in 8% of the samples.

Among the microorganisms resistant to the main antimicrobials used in the clinic, Gram-positive bacteria were found in 20% of clinical isolates, of which 50% were *Staphylococcus aureus* (*S. aureus*), mainly methicillin-resistant *S. aureus* (MRSA, 70%). Furthermore, among the coagulase-negative *Enterococcus* and *Staphylococcus* (coagulase-negative S.), two clinical isolates of vancomycin-resistant *Enterococcus* (VRE) and two methicillin-resistant *S. coagulase*-negative (MRSCON) isolates were identified. Regarding Gram-negative bacteria, 25 clinical isolates were identified, highlighting enterobacteria resistant to third- and fourth-generation cephalosporins (48% of enterobacteria), followed by *Pseudomonas aeruginosa* (*P. aeruginosa*) resistant to carbapenems (35% of *P. aeruginosa* isolates). *Acinetobacter baumannii* (*A. baumannii*) resistant to third-generation cephalosporins and carbapenems and *Stenotrophomonas maltophilia* (*S. maltophilia*) resistant to sulfamethoxazole and trimethoprim corresponded to 50% and 100% of these species, respectively.

It is noteworthy that 40% (56) of the 140 patients who developed sepsis had the first negative culture results. In these patients, the presence of microorganisms was detected and clinically characterized when sepsis was in full development. Empirical antibiotic treatment was initiated mostly with broad-spectrum drugs such as carbapenems (imipenem, meropenem), third- and fourth-generation cephalosporins, and vancomycin.

### 3.3. Platelet Immunophenotyping (Platelet Activation)

The basal level of expression of P2Y12, CD62P, CD41, and CD61 antigens on the surface of platelets showed a significant increase (*p* < 0.01) between platelets of patients before admission to the ICU and after the development of sepsis (Table 2). This difference was also found in relation to platelets from healthy volunteers (negative control).

### 3.4. Real-Time Expression of miRNAs miR-127 and miR-320a in Platelets

**Figure 1:**
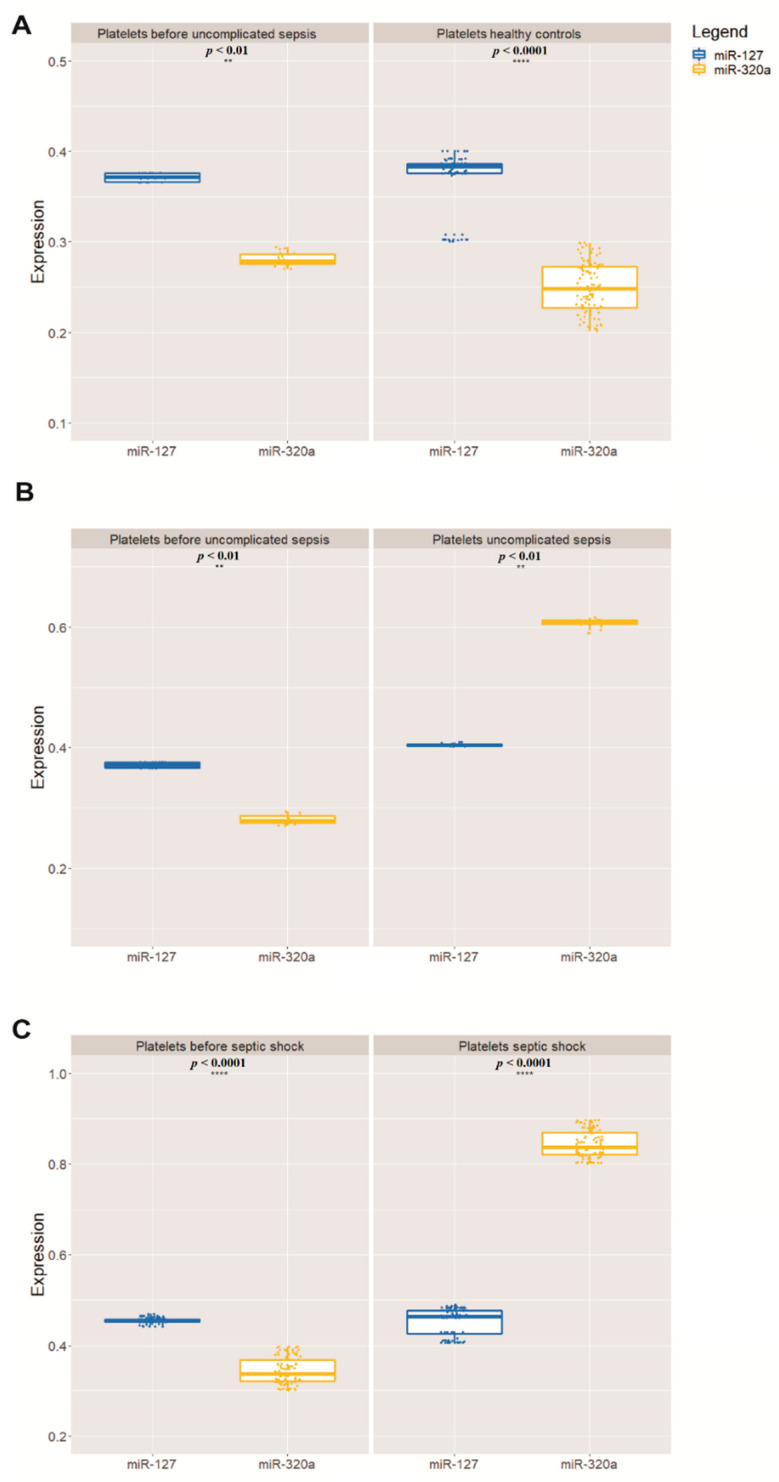
Relative mean expression of miRNAs quantitative PCR (qPCR) analysis (**A**). The Wilcoxon paired-sample test was applied to estimate the significance of expression of miR-127 and miR-320a in relation to miR-191 (**B**), which has been used as an internal control for validation analysis (** *p* < 0.01, **** *p* < 0.0001) (**C**). In all graphs, the X-axis represents the storage time of the PCs and the Y-axis represents the 100 PC units.

**Figure 2:**
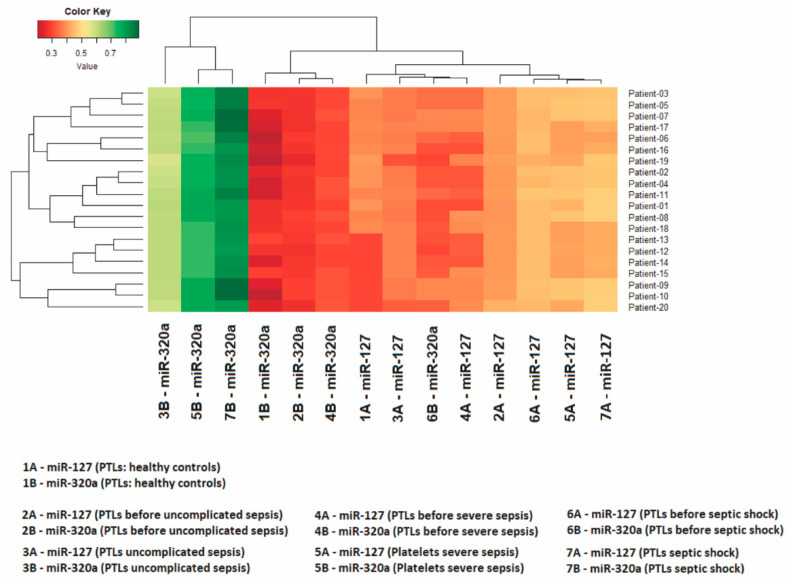
The heatmap shows expression levels in 20 patients. The Z-score was the metric applied to infer the clustering of the heatmap. Gradients with a red tendency represent miRNAs with a lower Z-score and gradients with a green tendency are those with a higher Z-score.

In all 100 volunteers who donated healthy platelets, as expected, the highest quantity was that of miR-127 in relation to miR-320a. Only the 140 patients who developed sepsis had the relative quantity of these miRNAs inverted—that is, the quantity of miR-320a was greater than that of miR-127 (Table 2, Figure 1). The relationship between the expression levels of these two miRNAs is shown in the heatmap in Figure 2, made for 20 patients who developed sepsis.

Importantly, the quantitative change in miRNAs was significant (*p* < 0.01) when comparing the values of patients who developed the three different types of sepsis versus the values of the same patients before sepsis and the values of healthy volunteers. The change in miR-127 and miR-320a also coincided with the development of thrombocytopenia, as the 140 patients who developed sepsis had platelets below 50,000/µL and increased procalcitonin (above 2.0 ng/mL).

## 4. Discussion

Sepsis is a clinical syndrome characterized by the presence of circulating microbial endotoxins and oxidative stress [13]. As sepsis is one of the primary diseases for which it is difficult to make a definitive diagnosis in emergency services, healthcare professionals face the problem of determining where these patients should be followed [14].

Platelets adhere and aggregate at sites of vascular injury to form a plug, which, together with coagulation components, protects the vessel’s integrity and prevents bleeding. Although platelets may have a beneficial role in the initial host response, platelet activation during sepsis contributes to the development of complications such as disseminated intravascular coagulation, multiple organ failure, acute lung injury, and acute kidney injury [15].

Thrombocytopenia is a strong marker of negative prognosis in patients with sepsis due to the low quality and/or consumption of platelets in these patients [16]. A low MPV in the blood may indicate a problem with platelet production (hyperproliferative thrombocytopenia), while a high MPV may indicate greater destruction (destructive thrombocytopenia). Patients with septic shock evaluated in this study had high hematological failure, showing a positive correlation between thrombocytopenia and sepsis. Our results corroborate those of the study by Mangalesh et al. [17] in which a high MPV and low platelet count were significantly associated with sepsis severity and mortality.

Elevated procalcitonin prohormone supported the diagnosis of sepsis, as its elevation may be associated with an inflammatory activity related to sepsis. In practice, it is believed that high values are indicative of more severe infectious processes [18,19].

To start appropriate therapy as soon as possible, it is important to identify the pathogen and perform antimicrobial resistance testing. The effectiveness of antibiotic treatment must be continuously monitored [20]. During infection, pathogens and their products influence the platelet response and can even be toxic. However, platelets can detect and engage bacteria and viruses to aid in their removal and destruction. Platelets contribute to host defense through several mechanisms, including the formation of immune complexes and the internalization of pathogens, and later being targeted for removal. These processes, such as the expression of surface receptors and the nature of platelet function in general, play important roles in pathogen detection [21].

Platelets modulate hemostasis and cellular responses through interactions with immune cells via secretion of immunomodulators and cell–cell interactions. The P2Y12 receptor mediates ADP-induced aggregation and secretion in platelets [22]. Clinical studies suggest that P2Y12 platelet inhibitors reduce sepsis mortality, although the underlying mechanisms have not been clearly defined in vivo. The P2Y12 inhibitors can improve sepsis survival by suppressing systemic inflammation and its prothrombotic effects [23].

The expression of CD62P on the membrane surface is considered an important indicator of platelet activation [4,24,25], and previous studies indicate that increased expression of CD62P on platelets is associated with damage to these cells [26,27]. Differences in platelet activation related to the quantity of soluble CD62P were observed among patients suffering from sepsis and hematologic malignancies. CD62P evaluation may be beneficial in the primary prevention of multiple organ failure in patients with sepsis [28].

Patients with septic shock had higher levels of CD41 and CD61. Increased amounts of CD41 indicate its release by activated platelets and are correlated with an unfavorable outcome [29]. Our results in platelets from ICU patients showed increased levels of CD41 and CD61, which has also been observed in platelets infected by the dengue virus [30].

Table 2 shows that any quantitative change that represents a significant increase in biomarkers P2Y12, CD62P, CD41, and CD61 is indicative of the presence of microorganisms in the patients’ bodies. In this study, these four biomarkers were reliable for monitoring the occurrence of sepsis. That is, when an increased MFI of these membrane antigens in relation to baseline values (found in negative controls) was detected, it was a sign of a systemic infectious process.

During the course of sepsis, the white blood cell counts increased and the drop in the number of platelets was inversely proportional to the increase in the membrane antigens studied in this project. Leukocytes play a fundamental role in innate and adaptive immunity, wound healing, tumor surveillance, and tissue remodeling. Platelets therefore circulate in a quiescent state and were rapidly activated by invading pathogens and other stimuli [31]. Measurement of miRNAs (127 and 320a) revealed this phenomenon. The inflammatory damage that the platelet suffers during sepsis activates the purinergic receptor P2Y12 through ADP, which is present in platelets and T lymphocytes and, for this reason, blocking P2Y12 improves the sepsis outcome [32].

As platelets suffer storage damage or are activated by the presence of microorganisms, there is an initial process of the degradation of miRNAs that reduces their quantity. However, this process ceases, and some miRNAs may have their quantity increased as the cell damage or sepsis progresses, as RNA-editing enzymes such as RNase and RNA helicases increase the quantity of certain miRNAs by cleaving miRNA precursors (pre-miRNAs) into mature miRNAs in response to oxidative stress [8,10,33].

This hypothesis could explain the increased quantity of miR-320a in relation to miR-127 (Table 2), since precursors of miR-320a can be cleaved to produce greater numbers of this miRNA, as miR-320a has a regulatory capacity to inhibit protein translation in response to stress [34]. In this case, specifically, the stress would be due to the somatic infection caused by sepsis. Evidence supporting our hypothesis is that miR-320a is responsive to oxidative stress and may regulate glycolysis in clinical diseases that are associated with changes in energy supply; in this case, the level of miR-320a increases up to 100-fold by cleavage of the pre-miR-320a [35].

Additionally, musculoskeletal pain conditions have also been shown to increase circulating levels of miR-320a, which reveals that this miRNA is responsive to stress [36]. Cell culture analyses indicate that miR-320a expression is also modulated by cellular stress [37]. Hyperoxia also increases miR-320a levels in epithelial-cell-derived microvesicles [38]. The most important evidence comes from an in vitro assay that simulates the body’s natural environment, where the secretion of miR-320a was indicative of cell stress and activation of the inflammatory response [39]. To our knowledge, there is no association of inflammatory or non-inflammatory oxidative stress with miR-127 in the literature.

The quantity of miR-127 and miR-320a does not identify which microorganism is responsible for sepsis, and thus does not replace the need for blood cultures, but these miRNAs are sensitive to the platelet activation process and their measurement in ICU patients can indicate which patients have suffered disseminated intravascular coagulation, which is a common and life-threatening complication of sepsis [40].

This work opens the perspective for an experimental study where the induction of infection is measured and the change in the behavior of these epigenetic and immunological markers can be monitored, so that we can better understand the mechanism that produces this phenomenon in the platelets of individuals with sepsis.

The 56 patients who developed sepsis and who had first negative culture results support the use of miRNAs when there is a clinical suspicion of progression to sepsis. In these patients, the presence of microorganisms was detected and characterized only when sepsis was in full development. Our results are supported by the study by Gupta et al. [41], who reported that 28–49% of analyzed patients who developed severe sepsis had the first negative culture results for the presence of microorganisms.

## 5. Conclusions

We conclude that quantifying the expression of P2Y12, CD62P, CD41, and CD61 antigens on the platelet surface and miRNAs miR-127 and miR-320a in platelets from patients with suspected sepsis, combined with the available clinical and diagnostic parameters, is a valuable tool to detect the onset of sepsis.

## Figures and Tables

**Table 1:** Clinical features of patients admitted to the ICU.

Variables	n
Patients admitted to the ICU	200
Patients admitted to the ICU with sepsis	140 (70%)
Male gender	122 (61%)
Average age, years (range)	60.75 (28–89)
Average length of stay in the ICU for patients without sepsis (days)	5
Average length of stay in the ICU for patients with sepsis (days)	15.7
APACHE II score average	35
Type of ICU admission—n (%) *	
Medical admission	132 (66%)
Surgical admission	68 (34%)
Infection Site—n (%) *	
Pulmonary	91 (65%)
Abdominal	42 (30%)
Urinary bacteremia	7 (5%)
Main Comorbidities—n (%) *	
Neoplasms	112 (80%)
Diabetes mellitus	15 (10.7%)
Systemic arterial hypertension (PAH)	13 (9.3%)
Invasive Procedures—n (%) *,#	
Urinary catheterization	77 (55%)
Central vascular catheterization	116 (82.9%)
Bladder probe	70 (50%)
Tracheostomy	105 (75%)
Mechanical ventilation	111 (79.3%)

Legend: * n = 140 (patients with sepsis); # most patients received more than one invasive procedure.

**Table 2:** Parameters evaluated in platelets of patients and volunteers (controls).

Parameters	Healthy Controls	Before Uncomplicated Sepsis	In Uncomplicated Sepsis	Before Severe Sepsis	Severe Sepsis	Before Septic Shock	Septic Shock
Membrane Proteins Expression							
P2Y12	39.28 ± 1.51	38.94 ± 0.80	101.24 ± 3.27 *	40.87 ± 0.89	166.64 ± 20.34 *	42.40 ± 1.94	281.85 ± 24.89 *
CD62P	9.22 ± 1.30	18.39 ± 2.09	72.19 ± 3.36 *	22.69 ± 3.55	121.06 ± 5.30 *	22.78 ± 3.47	159.97 ± 6.27 *
CD41	98.87 ± 4.30	108.83 ± 4.62	233.83 ± 4.49 *	121.12 ± 4.82	315.39 ± 4.72 *	130.62 ± 4.67	462.04 ± 7.52 *
CD61	87.46 ± 2.09	91.25 ± 1.33	233.83 ± 4.49 *	94.31 ± 1.72	308.56 ± 1.42 *	95.51 ± 1.37	394.95 ± 13.68 *
miRNAs Expression							
miR-127	0.369 ± 0.03	0.371 ± 0.004	0.404 ± 0.002	0.359 ± 0.033	0.442 ± 0.024	0.454 ± 0.005	0.453 ± 0.030
miR-320a	0.249 ± 0.02	0.281 ± 0.007	0.606 ± 0.007	0.309 ± 0.007	0.756 ± 0.024	0.343 ± 0.028	0.843 ± 0.028

Legend: * *p* < 0.01 when comparing the values of the patients who developed the three different types of sepsis versus the values of the same patients before sepsis and in relation to the values of healthy controls.
